# Elevated red cell distribution width-to-albumin ratio is associated with prevalent lung cancer in the U.S. population: A cross-sectional study

**DOI:** 10.1097/MD.0000000000050572

**Published:** 2026-09-11

**Authors:** Liming Peng, Jianbo Zhang, Jian Jiang, Chunhua Nie, Bo Yang, Lihua Li

**Affiliations:** a Department of Oncology, Yichun People’s Hospital, Yichun, Jiangxi, China; b The First Clinical Medical College, Nanchang University, Nanchang, Jiangxi, China.

**Keywords:** lung cancer, NHANES, RAR, restricted cubic splines, subgroup analysis

## Abstract

Red blood cell distribution width-to-albumin ratio (RAR) has recently been suggested as a potential inflammatory biomarker in various malignancies. However, its role in lung cancer (LC) remains underexplored. Data utilized in this study were extracted from the National Health and Nutrition Examination Survey spanning 2003 to 2020. Associations between RAR and LC were explored using logistic regression and multivariable adjustment techniques. To investigate potential nonlinear trends, restricted cubic spline modeling was applied. The diagnostic capacity of RAR was further assessed through analysis of receiver operating characteristic curves. Subgroup analysis by race and sex was conducted to assess heterogeneity. All analyses were weighted to account for the sampling design. Through screening, a total of >42,746 respondents were included in our study, among whom 89 cases of LC were identified. After applying sample weighting, a notable link was identified between the RAR and higher odds of prevalent LC. Logistic regression analysis showed that, compared with the RAR < 0.287, the 0.287 to 0.310 category was associated with lower odds of prevalent LC, whereas the >0.341 category was associated with higher odds; the 0.310 to 0.341 category was not significantly associated with LC prevalence. Additionally, increased red blood cell distribution width (RDW) and reduced serum albumin (Alb) concentrations were independently associated with higher odds of LC prevalence. Restricted cubic spline analysis indicated a nonlinear relationship between Alb and LC, while both RDW and RAR showed linear associations. Subgroup analysis revealed that RAR had a consistent harmful trend among people of different genders, educational levels, and alcohol consumption levels. Receiver operating characteristic analysis confirmed that RAR, RDW, and Alb had modest discriminative ability for identifying prevalent LC cases in this cross-sectional population. RAR is significantly associated with LC prevalence in a nationally representative sample, suggesting a notable epidemiologic association that warrants further longitudinal investigation.

## 1. Introduction

Lung cancer (LC) is one of the most common and deadly cancers worldwide, accounting for an estimated 1.8 million deaths each year, as reported by the World Health Organization.^[1]^ Detecting the disease at an early stage is vital for enhancing patient survival, since early-stage diagnoses are typically linked to more favorable prognoses.^[2]^ However, the absence of noticeable symptoms in initial stages frequently leads to late diagnoses, highlighting the urgent need for more efficient and accessible screening strategies. In this context, blood biomarkers have emerged as important tools for noninvasive diagnosis. Among these, red blood cell distribution width (RDW) and albumin (Alb) are gaining attention for their potential in LC screening due to their simplicity and predictive capabilities.^[3,4]^ Importantly, both RDW and Alb are widely regarded as nonspecific biomarkers that reflect systemic inflammation, nutritional status, and overall physiological condition rather than disease-specific processes.

Numerous studies have highlighted the relevance of RDW and Alb as biomarkers in various diseases, including cancer. RDW, which reflects the variation in red blood cell size, has been shown to correlate with the inflammatory status and prognosis of cancer patients, including those with LC.^[5]^ Nevertheless, RDW elevations may occur in a broad range of conditions, such as chronic inflammatory states, nutritional deficiencies, and other comorbidities. Similarly, Alb, an important negative acute-phase protein, is frequently decreased in malignancies and has been linked to poor outcomes in LC.^[6]^ Reduced serum albumin may also reflect systemic inflammation, malnutrition, or comorbid conditions, rather than being directly caused by tumor biology. However, decreased Alb levels can also reflect systemic inflammation, malnutrition, or other chronic illnesses, underscoring its nonspecific nature. Recent studies have also demonstrated the utility of the red blood cell distribution width-to-albumin ratio (RAR) in predicting the severity and prognosis of various diseases.^[7,8]^ RAR has shown promise as an efficient screening tool in cardiovascular and oncological diseases.^[9,10]^ Because RAR integrates RDW and Alb, it may capture combined inflammatory and nutritional alterations; however, it likewise represents a nonspecific physiological signal rather than a disease-specific marker. Despite these findings, RAR should still be considered a nonspecific physiological indicator rather than a disease-specific biomarker.

Despite its success in other conditions, the role of RAR in LC screening remains underexplored. This research utilizes data from the National Health and Nutrition Examination Survey (NHANES) database, covering the period from 2003 to 2020, to explore the relationship between the RAR and LC. Because NHANES is a cross-sectional survey, the observed relationships should be interpreted cautiously, as reverse causation is possible – for example, the presence of LC or related systemic effects may influence RDW or Alb levels.^[11]^ By analyzing nationally representative data and conducting multivariable, spline, and subgroup analyses, this study aims to clarify whether RAR is associated with the prevalence of LC in the general population. These findings may provide preliminary epidemiological evidence regarding the potential role of RAR as an accessible biomarker associated with LC.

## 2. Materials and methods

### 2.1. Data source

This investigation analyzes data from the NHANES survey conducted between 2003 and 2020, which aims to offer a nationally representative sample of the U.S. civilian, noninstitutionalized population using a stratified sampling technique.^[12]^ The dataset comprises both demographic and clinical information, essential for analyzing the connections between lifestyle factors and health outcomes. The NHANES study received ethical approval from the National Center for Health Statistics, and all individuals involved gave their written consent for the inclusion of their data in the analysis. This study is a secondary analysis of publicly available de-identified data and was therefore exempt from additional ethical review. All analyses were conducted in accordance with relevant guidelines and regulations.^[13]^ Given the relatively low proportion of missing data and the absence of an apparent systematic pattern of missingness, the likelihood of substantial selection bias due to these exclusions is considered minimal.

### 2.2. Study population

The study population consisted of NHANES participants from the survey period spanning 2003 to 2020, divided into 11 phases, with 1 phase conducted every 2 years. Due to the worldwide health emergency brought on by the Coronavirus Disease 2019 outbreak, NHANES ceased its in-person data collection efforts in March 2020. Consequently, the data gathered between 2019 and March 2020 were integrated with information from the preceding and following periods to form a pre-pandemic dataset spanning from 2003 to 2020. This analysis included participants aged 20 and older during this period, all of whom provided information on their test indicators and cancer status. Following NHANES analytic guidelines, when multiple survey cycles were combined, new sampling weights were calculated by dividing the 2-year examination weight by the number of cycles included. Survey analyses were conducted using the R survey package^[14]^ with sampling weights, masked variance pseudo–primary sampling units, and masked variance pseudo-strata to account for the complex sampling design.^[15–19]^

### 2.3. Exposure and outcome

The primary variable of exposure in this study is the RAR, calculated using the formula: RAR = RDW (%)/Alb (g/L).^[20]^ Participants were classified into either the LC or non-LC group based on their self-reported LC status, as indicated in the survey questionnaire. First, participants were asked whether they had ever been told by a doctor or health professional that they had cancer or a malignant tumor (MCQ220); then they were asked what kind of cancer it was (MCQ230a).^[21]^

### 2.4. Covariates

This study accounted for several covariates, including educational attainment, alcohol intake, smoking history, hypertension, diabetes status, age, sex, and race.^[22]^ Alcohol consumption was assessed based on participants’ self-reported average number of drinks consumed on drinking days over the past year. Participants who had smoked at least 100 cigarettes in their lifetime were defined as having a history of smoking. Those with a smoking history were further categorized as former smokers or current smokers according to their current smoking status. Hypertension was identified by a previous diagnosis from a healthcare provider, and diabetes was determined based on whether a doctor had informed the participant of having the condition. The variable names and codes used in NHANES are recorded in Table S1, Supplemental Digital Content 1.

### 2.5. Statistical analysis

Categorical variables are expressed as weighted ratios using NHANES survey weights to reflect complex multi-stage sampling designs. We use weighted mean and standard deviation to describe continuous data and calculate frequency and percentage for categorical data.

To evaluate the relationship between RAR and LC, a multivariable logistic regression model was applied. The independent variables are the values of the 3, and they are divided into multiple groups according to the quartile method. Three different models were adopted: model 1: unadjusted model, establishing the original association between RAR features and LC; model 2: a partially adjusted model, incorporated variables such as age, gender, and race; and model 3: the fully adjusted model, included additional factors like educational background, alongside other key demographic variables.^[23]^ To further explore the associations of RDW and Alb with LC, separate analyses were conducted for each biomarker.

Restricted cubic spline (RCS) regression was applied to explore the potential nonlinear relationship between RAR and LC. Three knots were placed at the 10th, 50th, and 90th percentiles of the RAR distribution, and nonlinearity was assessed using Wald tests.^[24]^

The discriminative ability of RAR for LC was evaluated using the area under the receiver operating characteristic (ROC) curve (AUC). AUCs and 95% confidence intervals (CIs) were estimated using the DeLong method. Internal validation was performed using 5-fold cross-validation and bootstrap resampling (200 iterations), and model calibration was assessed using the Hosmer–Lemeshow goodness-of-fit test.^[25]^

Sensitivity analyses were conducted using alternative regression approaches, including survey-weighted Poisson regression, Firth penalized logistic regression, and complementary log–log models.^[26]^

Subgroup analyses were performed using survey-weighted logistic regression models with the exposure standardized using *z*-score transformation. Effect modification was assessed by including interaction terms with Wald tests.^[27]^

Our analysis takes into account the NHANES sampling framework, which includes stratified, clustered, and weighted elements to generate nationally representative estimates.^[28]^

### 2.6. Statistical significance

All analyses were performed using R software (version 4.4.1, https://www.r-project.org/). Statistical significance was determined using 2-tailed tests, with a *P* value threshold of .05. For each regression model, odds ratios (ORs) and 95% CIs were computed to evaluate the strength and precision of the associations.^[26]^

## 3. Results

### 3.1. Characteristics of participants

Statistical comparisons show notable differences between the LC and non-LC groups, as shown in Table 1. Similar associations were found in the RDW (Table S1, Supplemental Digital Content 1) and Alb groups (Table S2, Supplemental Digital Content 2). The LC cohort shows a higher average age and greater representation of non-Hispanic white individuals. Health problems such as hypertension and diabetes are more common among participants with LC, and the smoking rate is also higher.

**Table 1:** Baseline characteristics of adult NHANES participants with and without prevalent self-reported lung cancer.

Characteristics	Overall (n = 42,746)	Non-LC (n = 42,657)	LC (n = 89)	*P*
Gender (%)				
Female	22,026 (51.5)	21,988 (51.5)	38 (42.7)	.118
Male	20,720 (48.5)	20,669 (48.5)	51 (57.3)	
Age (mean [SD])	49.70 (17.94)	49.66 (17.93)	67.78 (11.86)	<.001
Race (%)				
Mexican American	6773 (15.8)	6772 (15.9)	1 (1.1)	<.001
Non-Hispanic Black	9084 (21.3)	9065 (21.3)	19 (21.3)	
Non-Hispanic White	18,439 (43.1)	18,384 (43.1)	55 (61.8)	
Other Hispanic	3898 (9.1)	3894 (9.1)	4 (4.5)	
Other race – including multi-racial	4552 (10.6)	4542 (10.6)	10 (11.2)	
Education (%)				
9–11th grade	5950 (13.9)	5936 (13.9)	14 (15.7)	.247
College graduate or above	9631 (22.5)	9619 (22.5)	12 (13.5)	
High school graduate	9921 (23.2)	9894 (23.2)	27 (30.3)	
<9th grade	4641 (10.9)	4631 (10.9)	10 (11.2)	
Some college or AA degree	12,603 (29.5)	12,577 (29.5)	26 (29.2)	
Alcohol (mean [SD])	2.73 (2.83)	2.73 (2.84)	2.20 (1.52)	.081
Hypertension (%)				
No	27,439 (64.2)	27,399 (64.2)	40 (44.9)	<.001
Yes	15,307 (35.8)	15,258 (35.8)	49 (55.1)	
Diabetes (%)				
Borderline	948 (2.2)	945 (2.2)	3 (3.4)	.001
No	36,354 (85.0)	36,291 (85.1)	63 (70.8)	
Yes	5444 (12.7)	5421 (12.7)	23 (25.8)	
Smoke (%)				
Former	10,451 (24.4)	10,387 (24.4)	64 (71.9)	<.001
Never	23,560 (55.1)	23,552 (55.2)	8 (9.0)	
Now	8735 (20.4)	8718 (20.4)	17 (19.1)	
RAR (mean [SD])	0.32 (0.05)	0.32 (0.05)	0.35 (0.05)	<.001

LC = lung cancer, NHANES = National Health and Nutrition Examination Survey, RAR = red blood cell distribution width-to-albumin ratio, SD = standard deviation.

**Figure 1.**
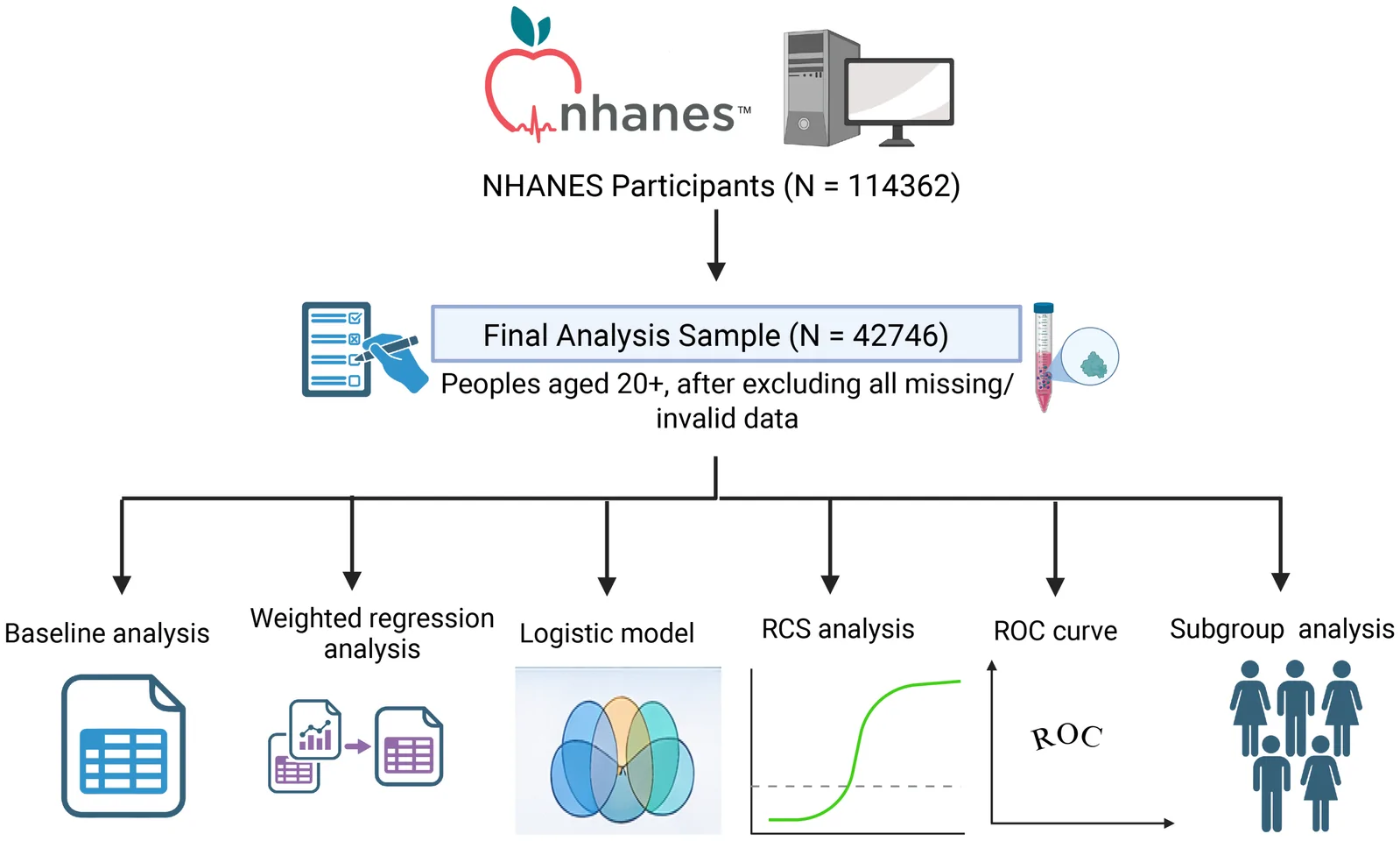
This study’s design and flowchart. NHANES = National Health and Nutrition Examination Survey, RCS = restricted cubic spline, ROC = receiver operating characteristic.

**Figure 2.**
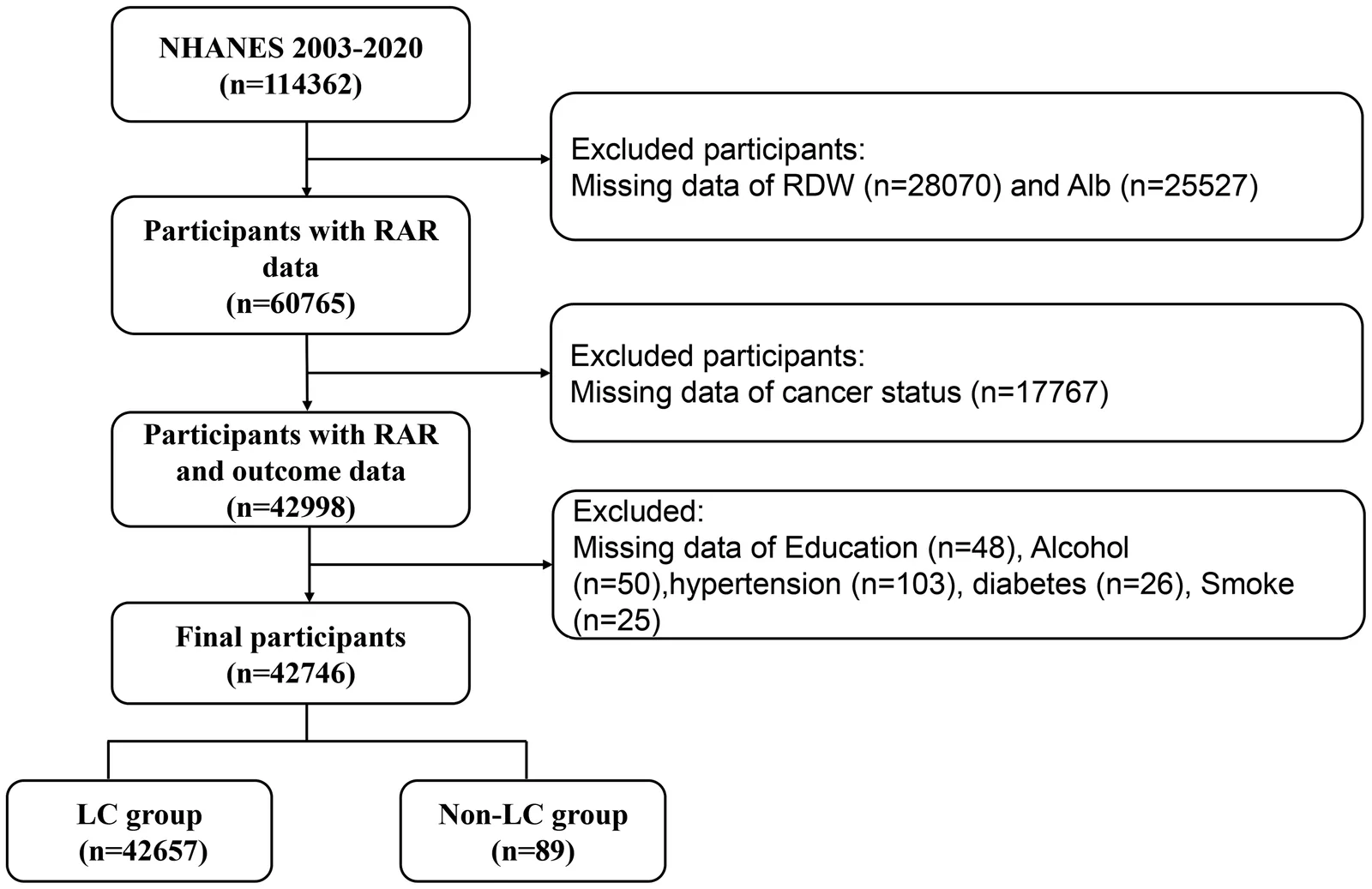
Flowchart of the sample selection from NHANES 2003–2020. Alb = albumin, LC = lung cancer, NHANES = National Health and Nutrition Examination Survey, RAR = red blood cell distribution width-to-albumin ratio, RDW = red blood cell distribution width.

Following the flowchart of this study (Fig. 1), the NHANES dataset from 2003 to 2020 comprised 114,362 participants, with 42,998 adults aged 20 or older eligible for inclusion. After excluding 252 individuals who lacked data on education, alcohol consumption, hypertension, diabetes, smoking, RDW, Alb, and RAR, the final analysis included 42,746 participants. Of these, 20,720 (48.5%) were male and 22,026 (51.5%) were female. In the overall sample, 89 participants (0.2%) were in the LC group, while 42,657 (99.8%) were in the non-LC group (Fig. 2). This sample corresponds to an estimated population of 169,973,056.3 individuals (Table 2). Similar associations were found in the RDW (Table S3, Supplemental Digital Content 3) and Alb groups (Table S4, Supplemental Digital Content 4).

**Table 2:** RAR baseline table of weighted data for lung cancer and the control group.

Characteristics	Overall (n = 169,973,056.3)	LC (n = 302,701.4)	Non-LC (n = 169,670,354.9)	*P* value
Gender (%)				.640
Female	87,857,946.5 (51.7)	147,215.3 (48.6)	87,710,731.2 (51.7)	
Male	82,115,109.7 (48.3)	155,486.1 (51.4)	81,959,623.6 (48.3)	
Age (mean [SD])	47.40 (16.95)	66.22 (12.06)	47.37 (16.94)	<.001
Race (%)				
Mexican American	14,280,335.1 (8.4)	802.5 (0.3)	14,279,532.6 (8.4)	<.001
Non-Hispanic Black	18,290,129.0 (10.8)	30,753.9 (10.2)	18,259,375.1 (10.8)	
Non-Hispanic White	115,111,102.8 (67.7)	248,971.3 (82.2)	114,862,131.5 (67.7)	
Other Hispanic	9,485,108.7 (5.6)	5340.8 (1.8)	9,479,767.9 (5.6)	
Other race	12,806,380.6 (7.5)	16,832.9 (5.6)	12,789,547.7 (7.5)	
Education (%)				
9–11th grade	17,501,506.2 (10.3)	46,622.7 (15.4)	17,454,883.4 (10.3)	.085
College graduate or above	49,272,735.6 (29.0)	52,509.2 (17.3)	49,220,226.3 (29.0)	
High school graduate	40,487,556.4 (23.8)	98,520.3 (32.5)	40,389,036.1 (23.8)	
<9th grade	9,431,404.9 (5.5)	30,275.4 (10.0)	9,401,129.5 (5.5)	
Some college or AA degree	53,279,853.3 (31.3)	74,773.8 (24.7)	53,205,079.4 (31.4)	
RAR (mean [SD])	0.31 (0.05)	0.34 (0.05)	0.31 (0.05)	<.001
Alcohol (mean [SD])	2.66 (2.66)	2.25 (1.60)	2.66(2.66)	.051
Hypertension (%)				.008
No	116,280,787.4 (68.4)	158,922.0 (52.5)	116,121,865.4 (68.4)	
Yes	53,692,268.9 (31.6)	143,779.4 (47.5)	53,548,489.4 (31.6)	
Diabetes (%)				
Borderline	3,413,173.3 (2.0)	15,443.5 (5.1)	3,397,729.8 (2.0)	<.001
No	150,451,778.5 (88.5)	213,493.4 (70.5)	150,238,285.1 (88.5)	
Yes	16,108,104.5 (9.5)	73,764.5 (24.4)	16,034,340.0 (9.5)	
Smoke (%)				<.001
Former	42,469,797.7 (25.0)	221,211.6 (73.1)	42,248,586.1 (24.9)	
Never	92,941,291.5 (54.7)	24,907.8 (8.2)	92,916,383.8 (54.8)	
Now	34,561,967.0 (20.3)	56,582.1 (18.7)	34,505,385.0 (20.3)	

LC = lung cancer, RAR = red blood cell distribution width-to-albumin ratio, SD = standard deviation.

Missingness for RDW and Alb was minimal, and participants with missing values were excluded during data preprocessing. Comparisons between included and excluded participants suggested no substantial differences in key demographic characteristics, indicating that the exclusion criteria were unlikely to introduce major selection bias.

### 3.2. LC prevalence assessment based on the RAR model

The RAR values were analyzed as continuous variables and classified into 4 quartiles: *Q*1 (<0.287), *Q*2 (0.287–0.310), *Q*3 (0.310–0.341), and *Q*4 (>0.341). The covariable-adjusted regression model revealed a significant overall association between the exposure groups. Notably, individuals in the *Q*2 range had lower odds of prevalent LC compared to those in *Q*1. The ORs for the 3 models were 0.24 (*P* = .17), 0.16 (*P* < .01), and 0.16 (*P* < .01), respectively. However, when RAR exceeded the *Q*3 range, a reversal of the continuous protective trend was observed, with RAR showing a harmful trend within the *Q*4 range (Table 3).

**Table 3:** The quartile feature method multiple regression model verifies the variable relationship.

Character	Model 1		Model 2		Model 3	
	95% CI	*P*	95% CI	*P*	95% CI	*P*
RAR						
*Q*1	Ref	–	Ref	–	Ref	–
*Q*2	0.26 (0.09–0.80)	.02	0.26 (0.08–0.80)	.02	0.25 (0.08–0.77)	.02
*Q*3	1.65 (0.70–3.96)	.26	1.63 (0.67–3.96)	.28	1.46 (0.59–3.62)	.41
*Q*4	3.90 (1.94–7.87)	<.01	3.93 (1.88–8.21)	<.01	3.32 (1.45–7.60)	<.01
RDW						
*Q*1	Ref	–	Ref	–	Ref	–
*Q*2	1.11 (0.44–2.84)	.81	1.12 (0.43–2.88)	.82	1.08 (0.42–2.79)	.88
*Q*3	1.34 (0.57–3.17)	.52	1.38 (0.58–3.31)	.47	1.24 (0.50–3.05)	.64
*Q*4	5.11 (2.56–10.22)	<.01	5.29 (2.57–10.92)	<.01	4.40 (2.01–9.66)	<.01
Alb						
≥35 g/L	Ref	–	Ref	–	Ref	–
<35 g/L	1.75 (0.57–5.41)	.33	1.81 (0.60–5.46)	.29	1.30 (0.43–3.91)	.64

Alb = albumin, CI = confidence interval, RAR = red blood cell distribution width-to-albumin ratio, Ref = reference, RDW = red blood cell distribution width.

Model 1: Unadjusted.

Model 2: Sex, age, race, and education level.

Model 3: Sex, age, race, education level, alcohol, hypertension, diabetes, and smoke.

Sensitivity analyses using alternative models (survey-weighted Poisson regression, Firth penalized logistic regression, and complementary log–log models) produced consistent estimates, indicating that the main findings were robust to different model specifications (Table S5, Supplemental Digital Content 5).

### 3.3. LC prevalence assessment based on RDW and Alb

The RDW value was analyzed as a feature and was also classified into 4 categories (*Q*1: <12.5, *Q*2: 12.5–13.1, *Q*3: 13.1–13.8, and *Q*4: >13.8). The covariable-corrected regression model shows a significant overall association among the exposure groups. However, individuals with RDW in the *Q*2 and *Q*3 intervals had higher odds of prevalent LC compared to those in *Q*1, but there was no statistical difference. When RDW exceeded the *Q*3 range, it was found that the RDW risk trend also continued, and RDW showed a harmful trend within the *Q*4 range. The ORs of the 3 models were 5.10 (*P* < .01), 5.29 (*P* < .01), and 4.40 (*P* < .01).

The Alb value was analyzed as a feature and was also classified into 2 categories (<35 and ≥35 g/L). The covariable-corrected regression model shows a significant overall association among the exposure groups. However, there was no statistical difference between the groups. The corresponding analytical findings are systematically documented in Table 3.

### 3.4. RCS analysis and ROC

**Figure 3.**
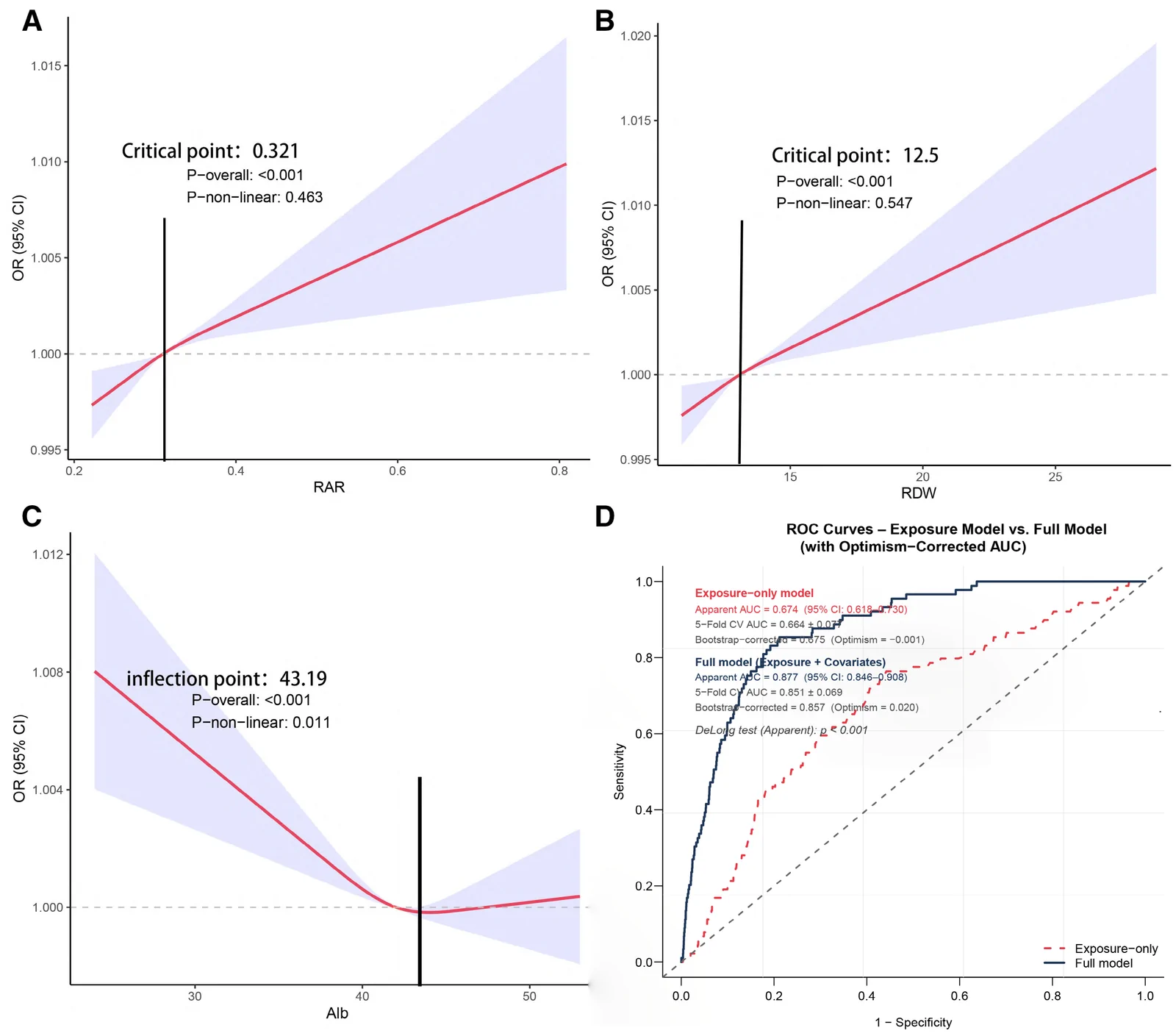
RCS and ROC curve analysis. (A) RAR and LC, (B) RDW and lung cancer, (C) Alb and lung cancer, and (D) multivariate ROC curve associated with lung cancer. Alb = albumin, AUC = area under the curve, CI = confidence interval, LC = lung cancer, OR = odds ratio, RAR = red blood cell distribution width-to-albumin ratio, RCS = restricted cubic spline, RDW = red blood cell distribution width, ROC = receiver operating characteristic.

The RCS analysis indicates that both RAR and RDW are overall associated with harmful effects (RAR: *P* overall <.001, *P* nonlinear = .463; RDW: *P* overall <.001, *P* nonlinear =.547). Critical thresholds were identified at 0.321 for RAR and 12.5 for RDW, beyond which the association shifts from protective to harmful. No significant nonlinear relationship was observed for either variable. In contrast, Alb exhibited a generally protective effect (*P* overall <.001), which began to attenuate and potentially reverse when values exceeded 43.19, with evidence of a mild nonlinear trend (*P* nonlinear =.011) (Fig. 3A–C).

ROC analysis was performed to explore the ability of RAR to distinguish individuals with prevalent LC. The exposure-only model yielded an AUC of 0.674 (95% CI: 0.618–0.730), whereas the multivariable model showed higher discriminative performance (AUC = 0.877, 95% CI: 0.846–0.908). Internal validation suggested minimal overfitting for the exposure-only model (5-fold cross-validation AUC = 0.664 ± 0.077; bootstrap-corrected AUC = 0.675), while the full model showed slightly reduced but still acceptable performance after correction (5-fold cross-validation AUC = 0.851 ± 0.069; bootstrap-corrected AUC = 0.857) (Fig. 3D). Calibration analysis showed that the exposure-only model had poor calibration (Hosmer–Lemeshow χ^2^(8) = 25.09, *P* = .001), whereas the multivariable model demonstrated better agreement between predicted probabilities and observed LC prevalence (Hosmer–Lemeshow χ^2^(8) = 7.42, *P* = .492) (Fig. S1, Supplemental Digital Content 6).

### 3.5. Subgroup analysis and forest plot visualization

Subgroup analyses were conducted according to gender, age, race, education level, alcohol consumption, hypertension, diabetes, and smoking status. The positive association remained generally consistent across most subgroups. Although the association appeared stronger among males, no significant interaction by gender was observed. Similarly, significant associations were present across age groups, race, and education categories, with no evidence of effect modification. Notably, alcohol consumption significantly modified the association (*P* for interaction =.012), with stronger associations observed at higher intake levels. In contrast, no significant interactions were identified for hypertension, diabetes, or smoking status (Table 4). Similar associations were found in the RDW (Table S6, Supplemental Digital Content 7) and Alb groups (Table S7, Supplemental Digital Content 8).

**Table 4:** A subgroup analysis was conducted on the association between RAR and LC.

Characteristics	Estimate	Std. error	*T* valuee	*P r*(> *t*)	Interaction 95% CI	Interaction *P* value
Gender						
Female	0.152	0.106	1.443	.151	1.50 (1.18–1.91)	<.001
Male	0.560	0.053	10.617	<.001		
Age						
≤60	0.217	0.105	2.067	.041	1.13 (0.88–1.45)	.340
>60	0.340	0.068	5.022	<.001		
Race						
Non-Hispanic Black	0.346	0.073	4.719	<.001	0.91 (0.73–1.13)	.434
Non-Hispanic White	0.251	0.076	3.312	.001		
Other Race	0.144	0.169	0.850	.387		
Education						
<9th grade	0.338	0.141	2.399	.018	0.91 (0.67–1.22)	.275
Some college or AA degree	0.239	0.058	4.149	<.001		
College graduate or above	0.514	0.163	3.162	.002		
Alcohol						
T1	0.220	0.078	2.817	.006	1.19 (0.98–1.46)	.151
T2	0.397	0.062	6.463	<.001		
T3	0.432	0.109	3.970	<.001		
Hypertension						
No	0.421	0.057	7.360	<.001	0.82 (0.71–0.96)	.012
Yes	0.225	0.060	3.727	<.001		
Diabetes						
No	0.329	0.051	6.408	<.001	0.83 (0.55–1.24)	.356
Yes	0.138	0.199	0.692	.490		
Smoke						
Never	0.239	0.203	1.182	.239	1.18 (0.77–1.78)	.607
Former	0.401	0.055	7.336	<.001		
Now	0.310	0.118	2.636	.009		

LC = lung cancer, *P r*(> *t*) = *T* value for the 2-tailed *t* test, RAR = red blood cell distribution width-to-albumin ratio, Std. = standard.

Estimate represents the regression coefficient (β); *T* value represents the Wald test statistic; interaction 95% CI represents the odds ratio (OR) and 95% confidence interval for the interaction term.

**Figure 4.**
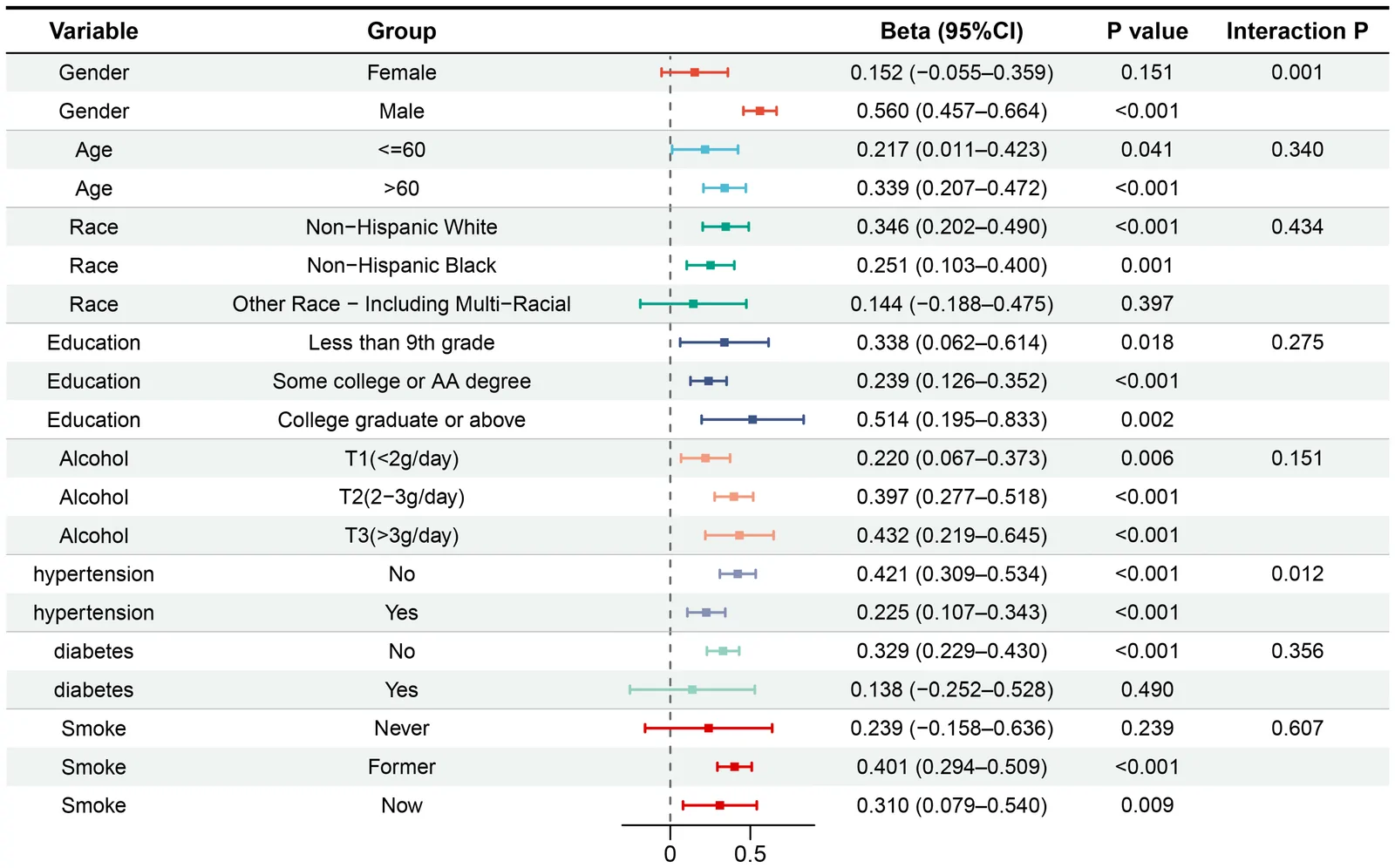
Beta values forest plot of subgroup analysis. Beta coefficients represent the change in the log odds of the outcome per 1 − standard‑deviation increase in RAR. CIs = confidence intervals, OR = odds ratio, RAR = red blood cell distribution width-to-albumin ratio.

**Figure 5.**
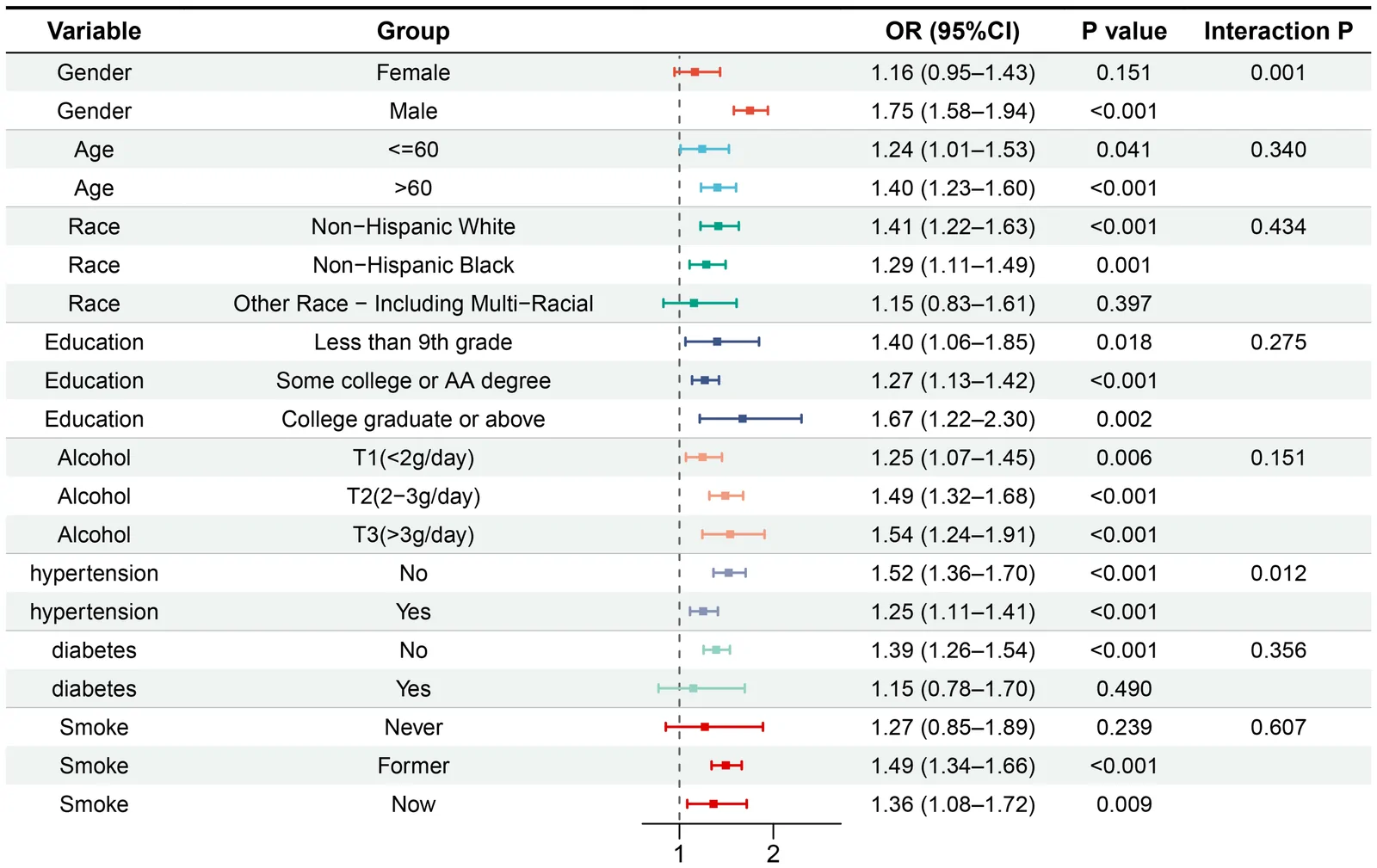
OR values forest plot of subgroup analysis. ORs and 95% CIs were estimated per 1 − standard-deviation increase in RAR. CIs = confidence intervals, OR = odds ratio, RAR = red blood cell distribution width-to-albumin ratio.

As shown in Figure 4, the forest plot displays the β values from the subgroup analysis, while Figure S2, Supplemental Digital Content 9 presents the ORs reflecting these subgroup differences. Similarly, forest plots of β values for RDW and Alb subgroups (Fig. 5) and OR plots (Fig. S3, Supplemental Digital Content 10) also indicated consistent findings.

**Figure 6.**
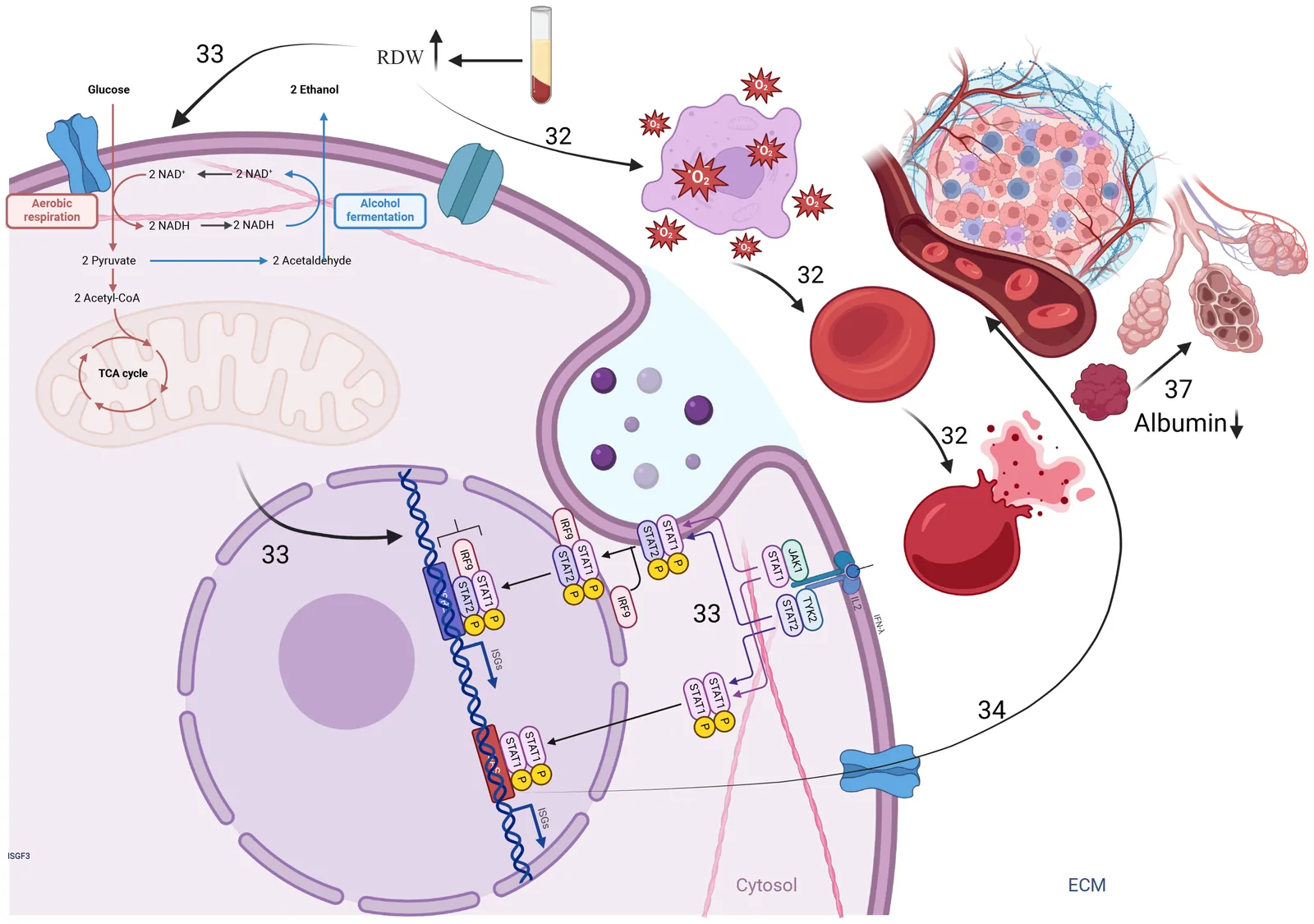
Conceptual schematic illustrating the proposed mechanisms underlying the association between RDW, albumin, and LC, based on previous literature. These pathways are hypothetical and were not directly tested in this study. LC = lung cancer, RDW = red blood cell distribution width.

Based on the current literature regarding the relationship between RDW, Alb, and cancer, the potential mechanisms linking RAR to LC prevalence are summarized in Figure 6.

## 4. Discussion

LC continues to rank among the top contributors to cancer mortality worldwide. While identifying the disease at an early stage significantly enhances the likelihood of survival, existing screening approaches often remain prohibitively expensive and out of reach for many.^[1,2]^ As a result, biomarkers like Alb, RDW, and the RAR have gained attention for their potential role in noninvasive risk stratification or epidemiological assessment of LC prevalence rather than standalone screening tools, although their combined use in association analyses has not been thoroughly studied.^[4,6]^ Previous studies have reported that hematologic and nutritional biomarkers are associated with LC outcomes. Elevated RDW has been linked to poorer survival and more advanced disease in LC patients, and RDW levels are often higher in individuals with non-small cell LC compared with healthy populations.^[29,30]^ In addition, low serum Alb has been identified as an independent predictor of recurrence and survival in early-stage LC.^[31]^ More recently, composite indicators integrating RDW and Alb, such as the RAR, have been proposed to reflect both systemic inflammation and nutritional status.^[32]^ Our findings highlight the potential association of RAR with LC prevalence, while also shedding light on the complex interactions between RAR, RDW, Alb, and other clinical variables.

To gain deeper insight into this relationship, it is essential to explore the physiological roles of RDW and Alb. RDW is a marker often linked to inflammation and systemic stress.^[33]^ Increased RDW levels are thought to reflect chronic inflammation, oxidative stress, and impaired erythropoiesis, all of which have been implicated in cancer development.^[34]^ High RDW levels have been observed in various cancers, including LC, as inflammatory processes in the tumor microenvironment may disrupt normal red blood cell production.^[35]^ Inflammatory cytokines, such as interleukin-6, are known to elevate RDW levels by affecting erythropoiesis and causing dysregulated red blood cell production.^[36]^ Alb is produced primarily by the liver and is widely recognized as a marker reflecting systemic inflammation, hepatic function, and nutritional well-being.^[37]^ Lower levels of Alb are frequently seen in cancer patients, reflecting malnutrition, hepatic dysfunction, and the catabolic state associated with malignancy.^[38]^ Alb acts as a negative acute-phase reactant, meaning its levels decrease during inflammation, which is commonly present in cancer.^[39]^ Taken together, RDW and Alb offer complementary perspectives: RDW captures inflammatory dysregulation, while Alb reflects metabolic and functional health.^[40]^ The ratio formed by combining these 2 markers, RAR, may thus serve as a comprehensive index capturing the interplay between inflammation and nutritional status – 2 key elements in cancer development and progression.^[41]^

Our study provides an in-depth analysis of RAR in LC prevalence, expanding on existing research that links RDW and Alb to cancer outcomes. While previous studies have explored RDW and Alb individually,^[42]^ our approach of combining these markers into RAR is novel and offers a more comprehensive view of LC prevalence. Rather than establishing prediction of future LC, our results suggest that higher RAR levels are associated with a greater prevalence of LC in this cross-sectional population. Unlike earlier studies, which often focused on small sample sizes or specific patient cohorts, our research, using data from a large, nationally representative cohort, offers more robust and generalizable results. These findings support a potential epidemiologic association between RAR and LC and suggest that RAR may serve as a hypothesis-generating marker for future longitudinal and mechanistic studies, particularly in resource-limited settings.

The RCS analysis offered further insights into the relationship between RAR, RDW, Alb, and LC prevalence. Alb exhibited a nonlinear relationship, with 2 critical points at 41.25 and 47.5, where the protective effect weakened. This suggests that while low Alb levels generally correlate with higher LC prevalence, very high Alb levels may signal a shift in the biological processes, possibly due to changes in protein metabolism or other underlying cancer mechanisms.^[43]^ In contrast, RDW and RAR showed a linear association with LC prevalence, with higher values linked to increased LC prevalence. This consistency indicates that RDW and RAR may offer a more straightforward prediction of LC prevalence compared to Alb, which requires more nuanced interpretation. The relatively high AUC observed in the multivariable model likely reflects the inclusion of established clinical and demographic predictors of LC rather than the independent predictive capacity of RAR alone. Therefore, RAR should be interpreted as an exploratory biomarker reflecting systemic inflammation and nutritional status rather than a standalone screening indicator.

The subgroup analysis revealed significant differences in the association between RAR and LC prevalence based on gender, hypertension status, and alcohol consumption. These observations indicate that the influence of RAR on LC susceptibility may differ across specific population subgroups defined by demographic or health-related characteristics, such as sex and comorbid conditions. Specifically, the relationship between RAR and LC prevalence appeared more pronounced in males, and lower LC prevalence was observed in individuals without hypertension. Alcohol consumption, on the other hand, appeared to exacerbate the prevalence of LC, which is consistent with previous studies showing the carcinogenic effects of alcohol. These results highlight the importance of considering subgroup differences when evaluating biomarker associations. The interaction between RAR and factors such as gender, hypertension, and alcohol consumption suggests that population heterogeneity may influence the observed associations. By identifying these subgroup effects, we can better understand the contexts in which RAR may be most effective as a predictive tool and refine its use in clinical practice.

This study has several strengths, including the use of a nationally representative NHANES dataset, which enhances the generalizability of these findings to the U.S. adult population. However, several limitations should be acknowledged. First, the cross-sectional design of NHANES precludes the establishment of temporal relationships or causality between RAR and LC. Second, LC status in NHANES is based on self-reported physician diagnosis, which may introduce misclassification bias. Third, although multiple covariates were adjusted for, residual confounding from unmeasured factors – such as environmental exposures, occupational hazards, genetic susceptibility, or detailed smoking history – cannot be excluded. Fourth, reverse causation is possible, as systemic inflammation and nutritional changes reflected by RDW and Alb may arise as consequences of underlying cancer rather than preceding it. Finally, the extremely low prevalence of LC in this dataset limits the potential clinical utility of these biomarkers for screening purposes, and the findings should therefore be interpreted as hypothesis-generating associations rather than evidence for clinical screening implementation. Future prospective cohort studies and mechanistic investigations are needed to clarify the temporal relationship between RAR and LC development.

## 5. Conclusion

Both elevated RAR and RDW levels were correlated with a heightened prevalence of self-reported LC, whereas higher Alb concentrations appeared to show an inverse association with LC prevalence. Among the markers evaluated, RAR exhibited the most robust discriminatory ability, particularly when integrated with prevalent self-reported LC. However, its epidemiological relevance warrants confirmation through future large-scale prospective studies.

## Acknowledgments

The authors appreciate all the public health workers and engineers who participated in the NHANES database, R software, and R language developers.

## Author contributions

**Conceptualization:** Liming Peng, Jianbo Zhang, Jian Jiang, Chunhua Nie, Bo Yang, Lihua Li.

**Data curation:** Liming Peng, Jianbo Zhang, Jian Jiang, Chunhua Nie, Bo Yang, Lihua Li.

**Formal analysis:** Liming Peng, Jianbo Zhang, Jian Jiang, Chunhua Nie, Bo Yang, Lihua Li.

**Investigation:** Liming Peng, Lihua Li.

**Methodology:** Liming Peng, Lihua Li.

**Project administration:** Liming Peng, Lihua Li.

**Resources:** Liming Peng, Lihua Li.

**Software:** Liming Peng, Lihua Li.

**Supervision:** Liming Peng, Lihua Li.

**Validation:** Liming Peng, Lihua Li.

**Visualization:** Liming Peng, Lihua Li.

**Writing – original draft:** Liming Peng, Jianbo Zhang, Jian Jiang, Chunhua Nie, Bo Yang, Lihua Li.

**Writing – review & editing:** Liming Peng, Lihua Li.




















