# Supplementary material for: Elevated red cell distribution width-to-albumin ratio is associated with prevalent lung cancer in the U.S. population: A cross-sectional study
Source: Medicine (Baltimore). 2026 Sep 11;105(37):e50572. doi: 10.1097/MD.0000000000050572 (PMC13574384; doi:10.1097/MD.0000000000050572)
Supplement: Supplementary file 1 [file medi-105-e50572-s001.docx]

**Table S1** NHANES variable names and code for reproducibility.

| **Domain** | **Variable Name** | **Description / Notes** |
| --- | --- | --- |
| Demographics | RIAGENDR | Gender |
| Demographics | RIDAGEYR | Age in years |
| Demographics | RIDRETH1 | Race/Ethnicity |
| Demographics | DMDEDUC2 | Education level |
| Demographics | ALQ101 | Alcohol consumption |
| Laboratory | LBXRDW | Red blood cell distribution width (%) |
| Laboratory | LBXALB | Serum albumin (g/L) |
| Questionnaire | MCQ160B, SMQ040 | Smoking status |
| Questionnaire | BPQ020 | Hypertension diagnosis (Yes/No) |
| Questionnaire | DIQ010 | Diabetes diagnosis (Yes/No) |
| Outcome | MCQ220, MCQ230a | Self-reported lung cancer |
