# Supplementary material for: Elevated red cell distribution width-to-albumin ratio is associated with prevalent lung cancer in the U.S. population: A cross-sectional study
Source: Medicine (Baltimore). 2026 Sep 11;105(37):e50572. doi: 10.1097/MD.0000000000050572 (PMC13574384; doi:10.1097/MD.0000000000050572)
Supplement: Supplementary file 2 [file medi-105-e50572-s002.docx]

Table S2 RDW and demographic characteristics were statistically analyzed in both the cancer and non-cancer groups.

| Characteristics | Overall (n=42746) | Non-LC (n=42657) | LC  (n=89) | P |
| --- | --- | --- | --- | --- |
| Gender (%) |  |  |  |  |
| Female | 22026 (51.5) | 21988 (51.5) | 38 (42.7) | 0.118 |
| Male | 20720 (48.5) | 20669 (48.5) | 51 (57.3) |  |
| Age (mean (SD)) | 49.70 (17.94) | 49.66 (17.93) | 67.78 (11.86) | <0.001 |
| Race (%) |  |  |  |  |
| Mexican American | 6773 (15.8) | 6772 (15.9) | 1 (1.1) | <0.001 |
| Non-Hispanic Black | 9084 (21.3) | 9065 (21.3) | 19 (21.3) |  |
| Non-Hispanic White | 18439 (43.1) | 18384 (43.1) | 55 (61.8) |  |
| Other Hispanic | 3898 (9.1) | 3894 (9.1) | 4 (4.5) |  |
| Other Race - Including Multi-Racial | 4552 (10.6) | 4542 (10.6) | 10 (11.2) |  |
| Education (%) |  |  |  |  |
| 9-11th grade | 5950 (13.9) | 5936 (13.9) | 14 (15.7) | 0.247 |
| College graduate or above | 9631 (22.5) | 9619 (22.5) | 12 (13.5) |  |
| High school graduate | 9921 (23.2) | 9894 (23.2) | 27 (30.3) |  |
| Less than 9th grade | 4641 (10.9) | 4631 (10.9) | 10 (11.2) |  |
| Some college or AA degree | 12603 (29.5) | 12577 (29.5) | 26 (29.2) |  |
| Alcohol (mean (SD)) | 2.73 (2.83) | 2.73 (2.84) | 2.20 (1.52) | 0.081 |
| Hypertension (%) |  |  |  |  |
| No | 27439 (64.2) | 27399 (64.2) | 40 (44.9) | <0.001 |
| Yes | 15307 (35.8) | 15258 (35.8) | 49 (55.1) |  |
| Diabetes (%) |  |  |  |  |
| Borderline | 948 (2.2) | 945 (2.2) | 3 (3.4) | 0.001 |
| No | 36354 (85.0) | 36291 (85.1) | 63 (70.8) |  |
| Yes | 5444 (12.7) | 5421 (12.7) | 23 (25.8) |  |
| Smoke (%) |  |  |  |  |
| No | 23560 (55.1) | 23552 (55.2) | 8 (9.0) | <0.001 |
| Yes | 19186 (44.9) | 19105 (44.8) | 81 (91.0) |  |
| RDW (mean (SD)) | 13.32 (1.38) | 13.96 (1.32) | 13.32 (1.38) | <0.001 |

Abbreviations: RDW: Red blood cell distribution width; SD: Standard deviation; P: Probability Value; LC: Lung cancer.
