# Supplementary material for: Elevated red cell distribution width-to-albumin ratio is associated with prevalent lung cancer in the U.S. population: A cross-sectional study
Source: Medicine (Baltimore). 2026 Sep 11;105(37):e50572. doi: 10.1097/MD.0000000000050572 (PMC13574384; doi:10.1097/MD.0000000000050572)
Supplement: Supplementary file 4 [file medi-105-e50572-s004.docx]

Table S4 RDW baseline table of weighted data for lung cancer and the control group.

| Characteristics | Overall  (n= 169973056.3) | LC  (n= 302701.4) | Non-LC  (n= 169670354.9) | P-Value |
| --- | --- | --- | --- | --- |
| Gender (%) |  |  |  | 0.640 |
| Female | 87857946.5 (51.7) | 147215.3 (48.6) | 87710731.2 (51.7) |  |
| Male | 82115109.7 (48.3) | 155486.1 (51.4) | 81959623.6 (48.3) |  |
| Age (mean (SD)) | 47.40 (16.95) | 66.22 (12.06) | 47.37 (16.94) | <0.001 |
| Race (%) |  |  |  |  |
| Mexican American | 14280335.1 (8.4) | 802.5 (0.3) | 14279532.6 (8.4) | <0.001 |
| Non-Hispanic Black | 18290129.0 (10.8) | 30753.9 (10.2) | 18259375.1 (10.8) |  |
| Non-Hispanic White | 115111102.8 (67.7) | 248971.3 (82.2) | 114862131.5 (67.7) |  |
| Other Hispanic | 9485108.7 (5.6) | 5340.8 (1.8) | 9479767.9 (5.6) |  |
| Other Race | 12806380.6 (7.5) | 16832.9 (5.6) | 12789547.7 (7.5) |  |
| Education (%) |  |  |  |  |
| 9-11th grade | 17501506.2 (10.3) | 46622.7 (15.4) | 17454883.4 (10.3) | 0.115 |
| College graduate or above | 49272735.6 (29.0) | 52509.2 (17.3) | 49220226.3 (29.0) |  |
| High school graduate | 40487556.4 (23.8) | 98520.3 (32.5) | 40389036.1 (23.8) |  |
| Less than 9th grade | 9431404.9 (5.5) | 30275.4 (10.0) | 9401129.5 (5.5) |  |
| Some college or AA degree | 53279853.3 (31.3) | 74773.8 (24.7) | 53205079.4 (31.4) |  |
| RDW (mean (SD)) | 13.16 (1.26) | 13.92 (1.30) | 13.16 (1.26) | <0.001 |
| Alcohol (mean (SD)) | 2.66 (2.66) | 2.25 (1.60) | 2.66 (2.66) | 0.073 |
| Hypertension (%) |  |  |  | 0.006 |
| No | 116280787.4 (68.4) | 158922.0 (52.5) | 116121865.4 (68.4) |  |
| Yes | 53692268.9 (31.6) | 143779.4 (47.5) | 53548489.4 (31.6) |  |
| Diabetes (%) |  |  |  |  |
| Borderline | 3413173.3 (2.0) | 15443.5 (5.1) | 3397729.8 (2.0) | <0.001 |
| No | 150451778.5 (88.5) | 213493.4 (70.5) | 150238285.1 (88.5) |  |
| Yes | 16108104.5 (9.5) | 73764.5 (24.4) | 16034340.0 (9.5) |  |
| Smoke (%) |  |  |  | <0.001 |
| No | 92941291.5 (54.7) | 24907.8 (8.2) | 92916383.8 (54.8) |  |
| Yes | 77031764.7 (45.3) | 277793.6 (91.8) | 76753971.1 (45.2) |  |

Abbreviations: RDW: Red blood cell distribution width; LC: Lung cancer; SD: Standard Deviation; P-Value: Probability value.
