# Supplementary material for: Elevated red cell distribution width-to-albumin ratio is associated with prevalent lung cancer in the U.S. population: A cross-sectional study
Source: Medicine (Baltimore). 2026 Sep 11;105(37):e50572. doi: 10.1097/MD.0000000000050572 (PMC13574384; doi:10.1097/MD.0000000000050572)
Supplement: Supplementary file 5 [file medi-105-e50572-s005.docx]

Table S5 Alb baseline table of weighted data for lung cancer and the control group.

| Characteristics | Overall  (n= 169907424.7) | LC  (n= 302701.4) | Non-LC  (n= 169604723.4) | P-Value |
| --- | --- | --- | --- | --- |
| Gender (%) |  |  |  | 0.639 |
| Female | 87840649.6 (51.7) | 147215.3 (48.6) | 87693434.4 (51.7) |  |
| Male | 82066775.1 (48.3) | 155486.1 (51.4) | 81911289.0 (48.3) |  |
| Age (mean (SD)) | 47.41 (16.95) | 66.22 (12.06) | 47.37 (16.93) | <0.001 |
| Race (%) |  |  |  |  |
| Mexican American | 14264223.7 (8.4) | 802.5 (0.3) | 14263421.2 (8.4) | <0.001 |
| Non-Hispanic Black | 18275762.6 (10.8) | 30753.9 (10.2) | 18245008.7 (10.8) |  |
| Non-Hispanic White | 115088892.3 (67.7) | 248971.3 (82.2) | 114839921.0 (67.7) |  |
| Other Hispanic | 9474327.1 (5.6) | 5340.8 (1.8) | 9468986.4 (5.6) |  |
| Other Race | 12804219.0 (7.5) | 16832.9 (5.6) | 12787386.1 (7.5) |  |
| Education (%) |  |  |  |  |
| 9-11th grade | 17487452.0 (10.3) | 46622.7 (15.4) | 17440829.3 (10.3) | 0.085 |
| College graduate or above | 49269060.4 (29.0) | 52509.2 (17.3) | 49216551.2 (29.0) |  |
| High school graduate | 40479678.4 (23.8) | 98520.3 (32.5) | 40381158.1 (23.8) |  |
| Less than 9th grade | 9429111.3 (5.5) | 30275.4 (10.0) | 9398835.9 (5.5) |  |
| Some college or AA degree | 53242122.6 (31.3) | 74773.8 (24.7) | 53167348.8 (31.3) |  |
| Alb (mean (SD)) | 42.44 (3.52) | 41.12 (4.03) | 42.44 (3.51) | 0.009 |
| Alcohol (mean (SD)) | 2.66 (2.66) | 2.25 (1.60) | 2.66 (2.66) | 0.051 |
| Hypertension (%) |  |  |  | 0.008 |
| No | 116247069.2 (68.4) | 158922.0 (52.5) | 116088147.3 (68.4) |  |
| Yes | 53660355.5 (31.6) | 143779.4 (47.5) | 53516576.1 (31.6) |  |
| Diabetes (%) |  |  |  |  |
| Borderline | 3397017.5 (2.0) | 15443.5 (5.1) | 3381574.0 (2.0) | 0.001 |
| No | 150402302.7 (88.5) | 213493.4 (70.5) | 150188809.3 (88.6) |  |
| Yes | 16108104.5 (9.5) | 73764.5 (24.4) | 16034340.0 (9.5) |  |
| Smoke (%) |  |  |  | <0.001 |
| Former | 42462017.0 (25.0) | 221211.6 (73.1) | 42240805.4 (24.9) |  |
| Never | 92892626.7 (54.7) | 24907.8 (8.2) | 92867718.9 (54.8) |  |
| Now | 34552781.1 (20.3) | 56582.1 (18.7) | 34496199.0 (20.3) |  |

Abbreviations: Alb: Albumin; LC: Lung cancer; SD: Standard Deviation; P-Value: Probability value.
