# Supplementary material for: Elevated red cell distribution width-to-albumin ratio is associated with prevalent lung cancer in the U.S. population: A cross-sectional study
Source: Medicine (Baltimore). 2026 Sep 11;105(37):e50572. doi: 10.1097/MD.0000000000050572 (PMC13574384; doi:10.1097/MD.0000000000050572)
Supplement: Supplementary file 6 [file medi-105-e50572-s007.docx]

**Table S6** A subgroup analysis was conducted on the association between RDW and LC.

| **Characteristics** | **Estimate** | **Std. Error** | **T-value** | **P r(>\|t\|)** | **Interaction P-value** |
| --- | --- | --- | --- | --- | --- |
| **Gender** |  |  |  |  |  |
| Female | 0.144 | 0.053 | 2.721 | 0.007 | 0.001 |
| Male | 0.377 | 0.045 | 8.334 | <0.001 |  |
| **Age** |  |  |  |  |  |
| <=65 | 0.214 | 0.042 | 5.103 | <0.001 | 0.904 |
| >65 | 0.205 | 0.070 | 2.947 | 0.004 |  |
| **Race** |  |  |  |  |  |
| Mexican American | 0.341 | 0.028 | 12.230 | <0.001 | 0.014 |
| Non-Hispanic Black | 0.013 | 0.126 | 0.101 | 0.920 |  |
| Non-Hispanic White | 0.296 | 0.054 | 5.526 | <0.001 |  |
| Other Hispanic | 0.212 | 0.089 | 2.392 | 0.018 |  |
| Other Race - Including Multi-Racial | 0.079 | 0.194 | 0.407 | 0.685 |  |
| **Education** |  |  |  |  |  |
| Less than 9th grade | 0.188 | 0.040 | 4.656 | <0.001 | 0.128 |
| Some college or AA degree | 0.276 | 0.053 | 5.198 | <0.001 |  |
| College graduate or above | 0.349 | 0.073 | 4.778 | <0.001 |  |
| **Alcohol** |  |  |  |  |  |
| Q1 | 0.207 | 0.034 | 6.119 | <0.001 | 0.338 |
| Q2 | 0.320 | 0.043 | 7.364 | <0.001 |  |
| Q3 | 0.280 | 0.098 | 2.864 | 0.005 |  |
| **Hypertension** |  |  |  |  |  |
| No | 0.223 | 0.044 | 5.025 | <0.001 | 0.910 |
| Yes | 0.230 | 0.046 | 5.051 | <0.001 |  |
| **Diabetes** |  |  |  |  |  |
| No | 0.226 | 0.039 | 5.826 | <0.001 | 0.281 |
| Yes | 0.151 | 0.092 | 1.636 | 0.104 |  |
| Borderline | 0.369 | 0.068 | 5.395 | <0.001 |  |
| **Smoke** |  |  |  |  |  |
| No | 0.277 | 0.079 | 3.485 | 0.001 | 0.601 |
| Yes | 0.228 | 0.039 | 5.874 | <0.001 |  |

**Abbreviations:** Std. Error: Standard Error; P r(>|t|): T-value for the two-tailed t-test; P-value: Probability Value; RDW: Red blood cell distribution width; LC: lung cancer.

**Note**: Estimate represents the regression coefficient (β); T-value represents the Wald test statistic.
