# Supplementary material for: Elevated red cell distribution width-to-albumin ratio is associated with prevalent lung cancer in the U.S. population: A cross-sectional study
Source: Medicine (Baltimore). 2026 Sep 11;105(37):e50572. doi: 10.1097/MD.0000000000050572 (PMC13574384; doi:10.1097/MD.0000000000050572)
Supplement: Supplementary file 7 [file medi-105-e50572-s008.docx]

**Table S7** A subgroup analysis was conducted on the association between Alb and LC.

| **Characteristics** | **Estimate** | **Std. Error** | **T-value** | **P r(>\|t\|)** | **Interaction P-value** |
| --- | --- | --- | --- | --- | --- |
| **Gender** |  |  |  |  |  |
| Female | 0.026 | 0.061 | 0.419 | 0.676 | 0.001 |
| Male | -0.212 | 0.034 | -6.291 | <0.001 |  |
| **Age** |  |  |  |  |  |
| <=65 | -0.052 | 0.058 | -0.894 | 0.373 | 0.679 |
| >65 | -0.090 | 0.066 | -1.367 | 0.174 |  |
| **Race** |  |  |  |  |  |
| Mexican American | -0.194 | 0.008 | -24.023 | <0.001 | 0.191 |
| Non-Hispanic Black | -0.183 | 0.034 | -5.369 | <0.001 |  |
| Non-Hispanic White | -0.097 | 0.040 | -2.441 | 0.016 |  |
| Other Hispanic | -0.097 | 0.052 | -1.862 | 0.065 |  |
| Other Race - Including Multi-Racial | -0.017 | 0.084 | -0.203 | 0.840 |  |
| **Education** |  |  |  |  |  |
| Less than 9th grade | -0.083 | 0.205 | -0.403 | 0.688 | 0.915 |
| Some college or AA degree | -0.036 | 0.054 | -0.663 | 0.508 |  |
| College graduate or above | -0.085 | 0.074 | -1.144 | 0.255 |  |
| **Alcohol** |  |  |  |  |  |
| Q1 | -0.064 | 0.040 | -1.596 | 0.113 | 0.188 |
| Q2 | -0.197 | 0.052 | -3.751 | <0.001 |  |
| Q3 | -0.147 | 0.091 | -1.621 | 0.107 |  |
| **Hypertension** |  |  |  |  |  |
| No | -0.135 | 0.049 | -2.779 | 0.006 | 0.265 |
| Yes | -0.035 | 0.064 | -0.548 | 0.585 |  |
| **Diabetes** |  |  |  |  |  |
| No | -0.131 | 0.033 | -3.923 | <0.001 | 0.162 |
| Yes | 0.068 | 0.134 | 0.508 | 0.612 |  |
| Borderline | 0.016 | 0.071 | 0.228 | 0.820 |  |
| **Smoke** |  |  |  |  |  |
| No | 0.064 | 0.156 | 0.407 | 0.685 | 0.247 |
| Yes | -0.116 | 0.036 | -3.261 | 0.001 |  |

**Abbreviations:** Std. Error: Standard Error; P r(>|t|): T-value for the two-tailed t-test; P-value: Probability Value; Alb: Albumin; LC: lung cancer.

**Note:** Estimate represents the regression coefficient (β); T-value represents the Wald test statistic.
